# A 6-year study on the mortality dynamics of sprouts germinated on *Schima superba* after a severe ice storm in southern China

**DOI:** 10.3389/fpls.2023.1178007

**Published:** 2023-05-12

**Authors:** Yonghui Cao, Benzhi Zhou, Xiaoming Wang

**Affiliations:** Research Institute of Subtropical of Forestry, Chinese Academy of Forestry, Fuyang, China

**Keywords:** branch sprout diameter, damage type, decapitation, ice storm, leaning, mortality rate of sprout, *Schima superba*, uprooted

## Abstract

**Introduction:**

Natural disturbances modify forest structure by affecting regeneration dynamics and can change main ecosystem functions. An ice storm unusually took place in southern China in early 2008, which caused huge damage to forests. Resprouting of woody plants in a subtropical forest has received little attention. The role of survival time and mortality has been assessed for newsprouts after an ice storm.

**Methods:**

In this study, damage types, in addition to the annual number and mortality rates of sprouts for all tagged and sampled resprouted Chinese gugertree (*Schima superba* Gardner & Champ.) individuals more than or equal to 4 cm in basal diameter (BD), were monitored. A total of six plots (20 m × 20 m) wererecorded in a subtropical secondary forest dominated by *S. superba* in Jianglang Mountain, China. This investigation had been conducted for six consecutive years.

**Results:**

The results showed that the survival rates of the sprouts were dependent on the year they sprouted. The earlier the year they boomed, the lower the mortality. The sprouts produced in 2008 were of the highest vitality and survival rates. Sprouts of the decapitated trees exhibited a better survival rate than those of uprooted or leaning trees. Sprouting position also plays a role in regeneration. Sprouts at the basal trunks of uprooted trees and the sprouts at the upper trunksof the decapitated trees exhibited the lowest mortality. The relationship between the accumulative mortality rate and the average diameter of new sprouts isaffected by damage types.

**Discussion:**

We reported the mortality dynamics of sproutsin a subtropical forest after a rare natural disaster. This information could serve asa reference for the construction of a branch sprout dynamic model ormanagement of forest restoration after ice storms.

## 1 Introduction

Climate change has been changing the structure and functional components of forest ecosystems at an unprecedented speed (Hof et al., 2021; Achim et al., 2022; Molina et al., 2022; Montoro Girona et al., 2023a), which has affected the function of forests on carbon fixation (Pan et al., 2011; Ameray et al., 2021). The sustainability of this carbon sink is mainly influenced by the frequency and intensity of large extreme events (LEEs) and depends on how quickly damaged forests can recover their photosynthetic capacity (Sun et al., 2012). Currently, our knowledge is limited on the eco-physiological processes controlling post-LEE recovery of the forest photosynthetic capacity, which leads to huge uncertainties in the estimation of net exchanges of terrestrial carbon cycles at different scales. Disturbances in forests can damage mature trees and also create conditions necessary for the reconstruction of new tree groups while creating miniature habitats where new species can settle (Sun et al., 2012).

Natural disturbances such as snow/wind, fire, and insects modify forest structure and can change main ecosystem functions, including net primary production (NPP) (Seidl et al., 2011; Haber et al., 2020; Kwon et al., 2021; Lavoie et al., 2021; Martin et al., 2022; Aakala et al., 2023). An ice storm as a natural disturbance can have significant influences on tree architecture and forest ecosystems (Brommit et al., 2004; Pisaric et al., 2008; Weeks et al., 2009; Turcotte et al., 2012). The canopy gaps caused by damage from ice storms will have various direct and long-term impacts on the structure and species composition of forests, tree regeneration dynamics, resource availability of forests, and so on (Nyland et al., 2016; Li et al., 2018). Ice storms are common in East Asia (Liu et al., 2020), North America, and Central Europe (Rustad and Campbell, 2012; Nyland et al., 2016; Priebe et al., 2018). In some countries where ice storms severely influence forest dynamics, case studies mainly focus on broadleaved forests or coniferous forests in Europe and North America (Nyland et al., 2016; Priebe et al., 2018). Previous studies have mainly studied tree plantations in comparison with natural forests. In the subtropics of China, natural broadleaved forests and natural mixed stands of conifers and broadleaved trees are typical vegetation types. Although ice storms could influence evergreen broadleaved forests, only recently, the importance of ice storms on forest development has been recognized (Zhou et al., 2011; Sun et al., 2012).

Stand regeneration is a key process to ensure the persistence of forest ecosystems, and the high mortality rate of trees or seedlings will hinder the establishment of regeneration (Lavoie et al., 2021). Resprouting, as a key functional trait in plant ecology, is an effective means that allows woody plants to regain their lost biomass after a disturbance and serves as the basis for the persistence niche (Bellingham and Sparrow, 2000; Poorter et al., 2010; Lawes and Clarke, 2011; Pausas et al., 2015; Clarke et al., 2016; Pausas and Keeley, 2017). However, the research on branch resprouting in response to a disturbance like ice storms of one tree species needs to be strengthened. Many studies on resprouting focused on the efficiencies of sprout regeneration, while sprouting growth and regeneration dynamics were less reported (Masaka et al., 2000; Del Tredici, 2001; Montoro Girona et al., 2018). The intraspecific variabilities of resprouting (Beaudet et al., 2007; Moreira et al., 2012; Bravo et al., 2014) and the long-term track records for resprouting mortality and survival rates of different sizes of individuals for a single tree species to a similar disturbance were also reported and needed an increase (Żywiec and Holeksa, 2012).

A previous study showed that the sprouting regeneration ratio was associated with the damage degree (Masaka et al., 2000; Del Tredici, 2001). A rare opportunity to assess branch resprouting ability and its dynamics was made by a heavy ice storm that occurred in early 2008 in China (Zhou et al., 2011; Wang et al., 2016). The ice storm hit southern and central China, a primary region of China’s terrestrial carbon storage, from 10 January to 6 February 2008 (Zhou et al., 2011). In these studies, the types of damage to trees were classified into crown decapitated, stem broken, branch broken, bending, and uprooted. At the stand level, damages ranged from the loss of leaves to the destruction of the entire forest. Approximately 10% of China’s forests were damaged in the ice storm (Zhou et al., 2011; Sun et al., 2012). Forests composed of evergreen broadleaved trees and conifer species cover most of the subtropical regions of southern China (Liu et al., 2020). The Chinese guger tree (*Schima superba* Gardner & Champ.) is one of the dominant tree species in subtropical evergreen broadleaved forests in China (Da et al., 2004; Wang et al., 2007). It is shade-tolerant and its regeneration is sporadic (Da et al., 2004). This species is valued commercially for its timber and biological traits, which enable this species to resist fires and prevent fire spread. *Schima superba* heavily sprouts in the canopy layer. The ice storm in 2008 caused disastrous damages which resulted in 92.61% of the forest canopy gaps due to tree falls.

The literature regarding the relationships between regeneration, vigor of sprouts from ice storm-damaged trees, type of damage, and sprout mortality is scarce (Cao et al., 2020). To the best of our knowledge, there are no reports about branch sprout mortality rates on damaged trees during the period of recovery. Therefore, research regarding the resprouting vigor, mortality rates, and survival of *S. superba* after forest disturbances can be a key in the management of the species’ commercial and ecological interest. This study was conducted in a “natural laboratory,” a subtropical secondary forest formed after the heavy ice storm fall in southern China in 2008. Data were collected for six consecutive years. Three major damage types, stumps of uprooted, leaning, and decapitated trees, were classified. The annual resprouting rate, sprout mortality rate, and survival duration of *S. superba* were recorded. We aimed to 1) examine the effect of this ice storm on the regeneration ability and sprout vigor of *S. superba*, (2) examine the variance of sprout mortalities and sprout life spans post-disturbance, and 3) explore the correlation between the accumulative mortality rates and the average diameter of new sprouts. The information gathered had important practical and theoretical values in understanding and managing the recovery of forests and vegetation succession after ice storms (Sun et al., 2012; Cao et al., 2020; Liu et al., 2020).

## 2 Materials and methods

### 2.1 Study site

**Figure 1:**
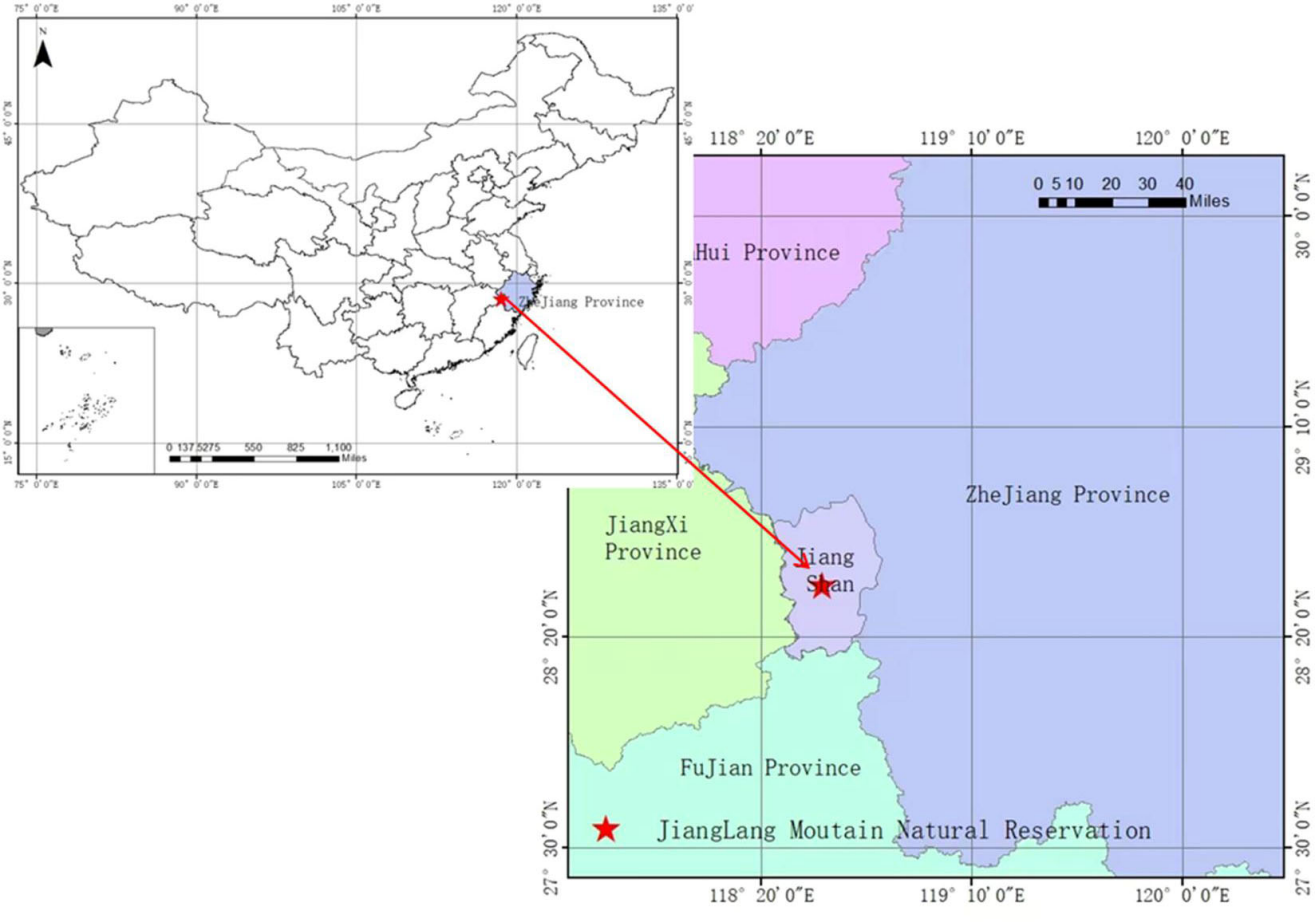
Location of the study area.

The study area is located in Mount Jianglang Nature Reserve (28°52′26″N, 118°48′37″E), Zhejiang Province, China (**Figure 1**). The altitudinal range, soil type, annual average temperature, annual average precipitation, natural vegetation, and so on in this study site were elaborated in our previous research (Cao et al., 2020). Below 900 m sea level, the vegetation of the mountain included forests of *S. superba*, Masson’s pine (*Pinus massoniana* Lamb.), Chinese fir [*Cunninghamia lanceolata* (Lamb.) Hook], Moso bamboo [*Phyllostachys edulis* (Carriere) J. Houzeau], secondary shrubs, and other vegetation types [see Cao et al. (2020)].

In the survey area where the ice storm disaster has caused significant damages, the dominant species of the secondary forests are *C. lanceolata*, *P. massoniana*, hardleaf oatchestnut [*Castanopsis sclerophylla* (Lindl.) Schott.], *Castanopsis fabri* (*Castanopsis fabri* Hance), and Oriental white oak [*Cyclobalanopsis glauca* (Thunb.) Oerst.] (Cao et al., 2020). The damaged forests selected for the permanent plot are located at an altitude height of 350 to 500 m and on a southwest or northwest slope at angles of 28° to 37°. The pedogenic materials featured are sedimentary rocks, and the soils are yellow loams in depth from 70 to 100 cm [see Cao et al. (2020)].

### 2.2 Method

#### 2.2.1 Plot and tree monitoring

A total of six plots (20 m × 20 m) were set up in April and May of 2008. General surveys were conducted on the types and degrees of damage to different species, including the angle of inclination of a damaged trunk relative to the original upright position (Cao et al., 2020). Montoro Girona et al. (2019) classified dead trees as standing dead, overturned, or broken. In this study, damages to *S. superba* trees were also categorized into eight types: undamaged, dead, uprooted, leaning, bending, decapitation, branch-broken, and stem-broken [see Cao et al. (2020)]. Trees with a basal diameter (BD) of more than or equal to 4 cm within each plot were tagged and investigated. The damage types, diameters at breast height (DBH), and basal diameters and heights of the tree stumps and crown widths were recorded in 2008 immediately after the ice storm. The forest has an average density of 2,400 trees per ha, a stand basal area of breast height of 27.25 m^2^ ha^−1^, and a mean tree diameter at breast height (DBH) of 11.9 cm in the stand with DBH varying up to 25 cm (**Table 1**) (Cao et al., 2020). There were approximately 69 trees of *S. superba* and 64 damaged trees of *S. superba* in each plot (**Table 1**).

**Table 1:** Information of the six sample plots and the growth traits of *Schima superba*.

Sample plots	Plot area (m^2^)	Slope aspect	Altitude (m)	Latitude	Longitude	Slope position	Stand density (stems·ha^−2^)	Mean DBH of the stand (cm)	Total basal area of breast height (m^2^·ha^−2^)	Number of *S. superba* (stems·ha^−2^)	Percentage of *S. superba* in the sample plot (%)	Mean DBH of *S. superba* (cm)
Plot 1	400	Western	447	28°31′8″	118°34′2″	Middle	2,800	11.72 ± 3.22	32.45	2,200	78.57	11.94 ± 3.09
Plot 2	400	Northeast	447	28°31′8″	118°34′2″	Middle	3,675	10.38 ± 3.02	33.50	3,200	87.07	10.24 ± 2.70
Plot 3	400	Northeast	447	28°31′8″	118°34′2″	Middle	2,950	10.18 ± 3.28	26.48	2,750	93.22	9.99 ± 3.10
Plot 4	400	North	370	28°31′8″	118°34′1″	Downhill	1,300	12.77 ± 4.55	18.67	450	34.62	14.60 ± 6.54
Plot 5	400	Southeast	370	28°31′8″	118°34′1″	Middle	1,725	15.23 ± 5.83	24.78	750	43.48	23.43 ± 4.40
Plot 6	400	North	380	28°31′8″	118°34′1″	Uphill	1,925	10.82 ± 4.02	27.65	1,025	53.25	10.20 ± 3.56

#### 2.2.2 Sampled trees and sprout growth measurement

To study the generation of sprouts, fallen trees (uprooted and leaning trees) and decapitated trees were selected for long-term monitoring. Thirty trees of each type were selected and tagged for the entire plots, and the DBH, BD, height of the stump, and the resprouting characteristics, including the total sprout number, new sprout number, BD of new sprouts, total length, and new increment of sprouts, were measured annually since mid-October of 2008 [for the method of sprout investigation along the trunk stem, see Cao et al. (2020)]. The early and later sprouting positions from each damaged trunk and all sprout living positions were recorded.

#### 2.2.3 Sprout mortality rate measurement

For the three damaged types of trees, the annual cumulative sprout mortality rate was calculated according to the number of annual new sprouts and the number of existing sprouts with the following formula:


$$M_{t}\left(\%\right)=\left(N_{i}-{\mathrm{Nt}}_{-i}\right)/N_{i}\times 100,i=0,1,\ldots ,4;t=1,2,\ldots ,5(t>i)$$

In the formula, *N* ***_i_*** represents the number of new sprouts germinated in the *i*-th year after the disaster, i.e., when *i* is 0; *N*_0_ represents the number of new sprouts that occurred in the disaster year (2008 year); and when *i* is 1, *N*_1_ represents the number of new sprouts that occurred in the second year after the disaster (2009), etc. *N* ***_t_***_−_ ***_i_*** represents the cumulative survival number of new sprouts in the *i*-th year after the restoration of *t* years. *M* ***_t_*** represents the cumulative mortality rate of new sprouts boomed in the *i*-th year after restoration of *t* years (i.e., when *i* = 0, *N* ***_i_*** represents the number of new sprouts in 2008, and so on. Only when *i* = 0, *t* = 1, at this time, *N* ***_t_***_−_ ***_i_*** = *N*_1_ represents the number of new sprouts in 2008 retained in 2009, and so on; at this time, *M* ***_t_*** represents the mortality rate of the new sprouts of 2008 in 2009).

#### 2.2.4 Data analysis

Statistical analyses were made using Microsoft Excel 2003 (11.0, Microsoft Corporation, WA, USA) and SPSS 16.0 (SPSS Corporation, Chicago, USA). One-way analysis of variance (ANOVA) was performed to compare the mortality rate of sprouts among the three damage types (Cui et al., 2022), and multiple comparisons (Tukey and Dunnett’s C methods) were used to analyze the significance of variance among the damaged types.

## 3 Results

### 3.1 Annual changes in the mortality rate of sprouts

**Figure 2:**
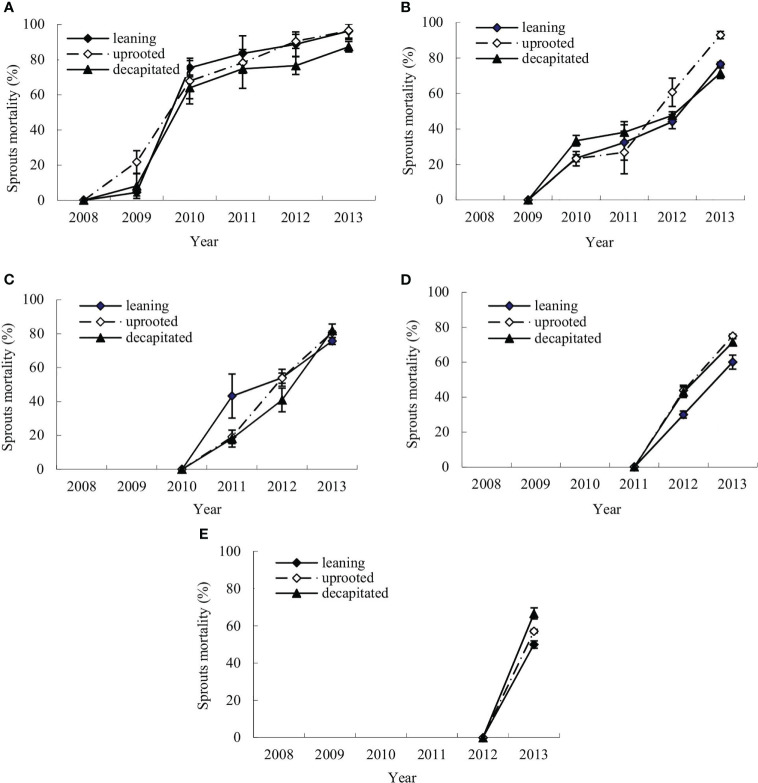
Annual changes of sprout mortality for new sprouts of each year of the damaged trees. **(A–E)** The means of mortality rates for the new sprouts per tree that were generated in 2008, 2009, 2010, 2011, and 2012, respectively, during recovery after the ice storm.

The mortality dynamics of the sprouts of *S. superba* were monitored after the ice storm. **Figures 2A–E** denote the means of annual cumulative mortality rates of the new sprouts of *S. superba* in each damage type. The annual cumulative sprout mortality rates were evaluated in the first (2008), second (2009), third (2010), fourth (2011), and fifth year (2012) after the ice storm. It was shown that the mortality rates of new sprouts that boomed each year on the damaged trees were increasing over time (**Figure 2**).

The new sprouts produced in the disaster year had a lower mortality rate in the next year after germination, from 4.48% ± 2.01% (leaning tree) and 8.11% ± 7.00% (decapitated tree) to 21.74% ± 6.43% (uprooted tree). The rate was rapidly increased in the third year and continuous in the following year with a maximum of 96.27% ( ± 7.47%) in the sixth year (**Figure 2A**). The sprouts that germinated in the second year showed a more rapid increase in mortality rate, from 23.21% ± 4.02% (uprooted tree) to 33.33% ± 3.51% (decapitated tree) in the next year (**Figure 2B**), and then increased slowly and reached 92.86% ± 2.11% (uprooted tree) in the fourth year. Similar to the sprouts produced in the early years, sprouts produced in the third year showed also an increase in mortality rate with time, and that rate was increased to 81.82% (decapitated tree) after 3 years from generation (**Figure 2C**). The sprouts that germinated in the fifth year were of the highest mortality rates in the next year after germination (**Figures 2D, E**). These results indicated that the earlier the sprouts germinated, the lower the mortality rate. Thus, the earlier sprouts germinated from damaged trees are of bigger contribution to regeneration. Comparing the three types of damaged trees, sprouts from decapitated trees were of the lowest mortality in the sixth year.

### 3.2 Sprout-germinated positions affect sprout survival rates

**Figure 3:**
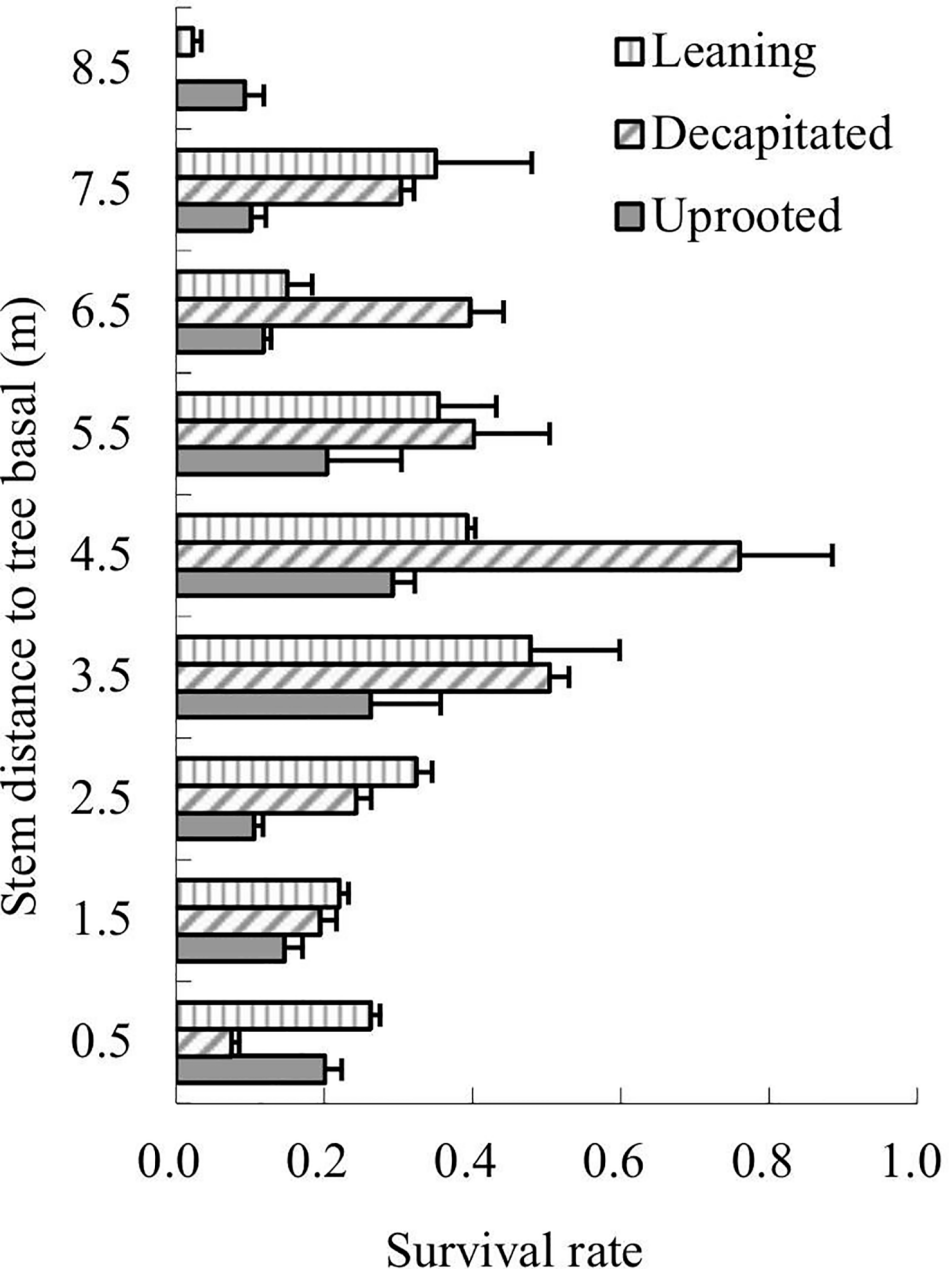
The survival rates of sprouts retained from 2009 to 2013. Sprout position on the three types of damaged trees was analyzed.

The survival rates of total sprouts germinated in the year 2009 of each type of the damaged trees were analyzed in 2013 according to sprout positions on the damaged trees (**Figure 3**). The results showed that sprouts of decapitated trees were of the highest survival rates while the uprooted trees were the lowest. For the decapitated trees, sprouts at the middle of the trunks exhibited the highest survival rate (>50%); for the leaning trees, sprouts from the middle or upper parts of the trunks were higher in survival rate; for uprooted trees, sprouts close to the trunk base or on the middle of the stems were higher (**Figure 3**). Thus, the decapitated trees recovered more rapidly, while the uprooted trees were the slowest in recovery.

**Figure 4:**
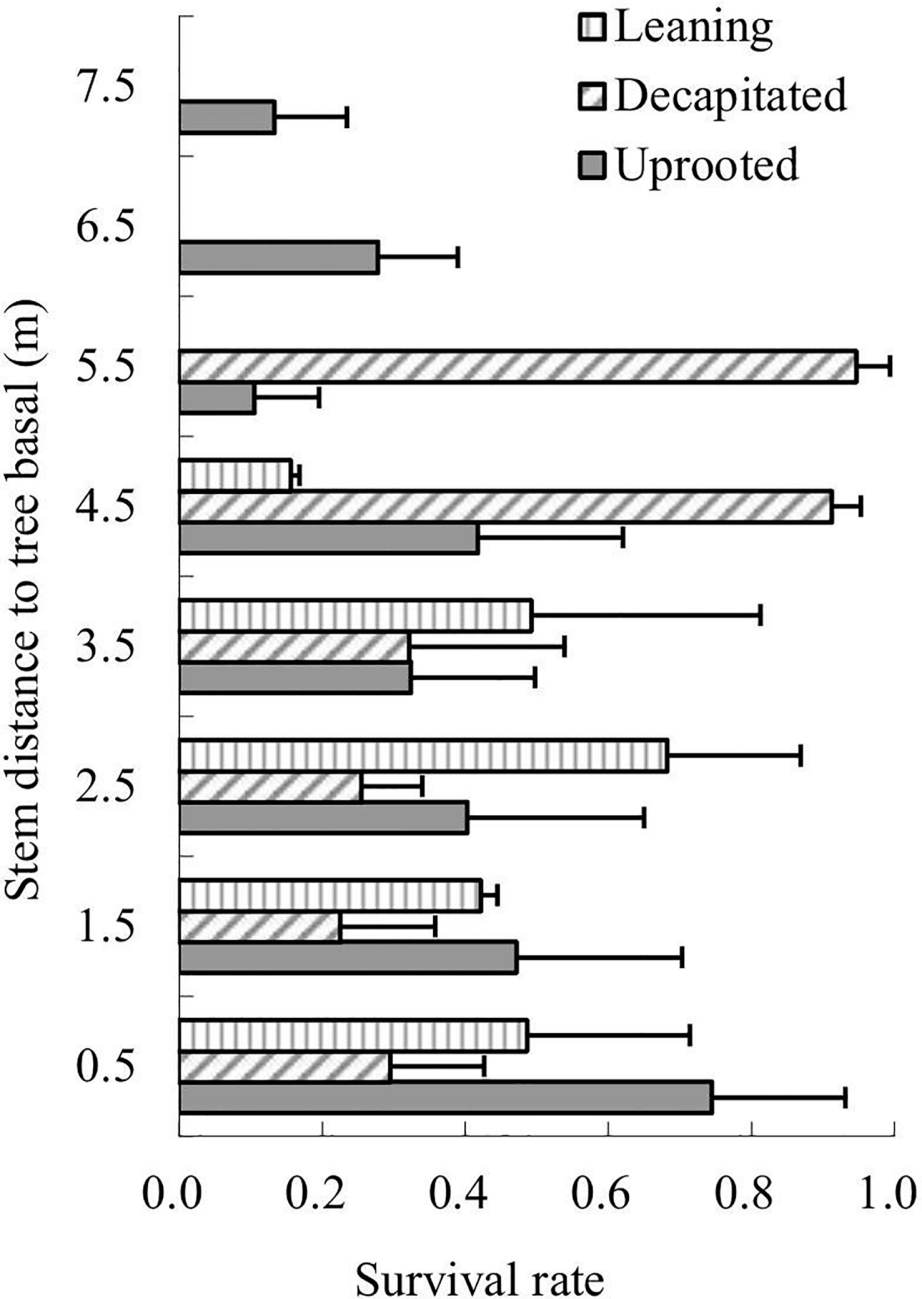
The survival rates of sprouts germinated in 2008 over 6 years of recovery.

The sprouts that germinated in the year 2008 were also analyzed. Over 6 years of recovery, the survival rates of sprouts of uprooted trees decreased along with the height of the sprouting positions; in contrast, the sprout survival rates of decapitated trees increased along with the height of the sprouting positions; sprouts on the lower parts of the trunks of leaning trees exhibited relatively similar survival rates (**Figure 4**). Thus, sprouts on the upper parts of the decapitated tree and sprouts on the base of uprooted trees were of the highest surviving vigor and could be kept to grow in order to enhance forest regeneration.

### 3.3 Relationship between sprout basal diameters and sprout mortality rates

**Figure 5:**
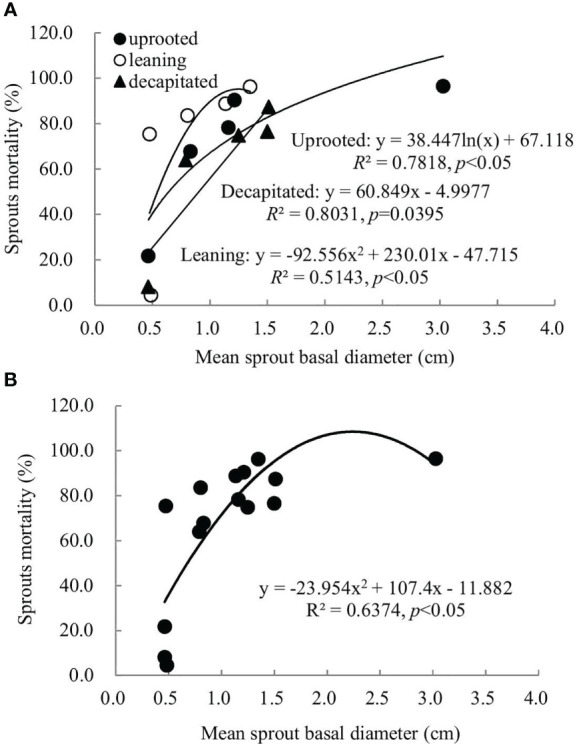
The relationship between the sprout basal diameters and the sprout mortality rates of sprouts germinated in 2008 was recorded over 6 years of recovery from damage. Sprouts of uprooted trees, leaning trees, and decapitated trees **(A)** and the total of all damaged trees **(B)** were analyzed, respectively.

A non-linear correlation between the accumulative mortality rates and the average diameters of sprouts was found when we analyzed all damaged trees, while trees classified to each damaged type exhibited a linear or non-linear correlation (**Figure 5**). Thus, the correlation between these two factors above is dependent on the damaged types of *S. superba*. The correlation was a significant logarithmic function relationship for sprouts of the uprooted trees, a polynomial relationship for sprouts of the leaning trees, and a significant linear positive correlation for decapitated ones, respectively (*p*< 0.05). For all damaged trees, the average diameters of the newly sprouted sprouts in the year 2008 had a significant polynomial correlation with the cumulative mortality rates during recovery from damage (**Figure 5**, *p*< 0.05). This indicates that the cumulative mortality rates increase along with the diameter of the sprouts germinated in 2008 year by year.

**Figure 6:**
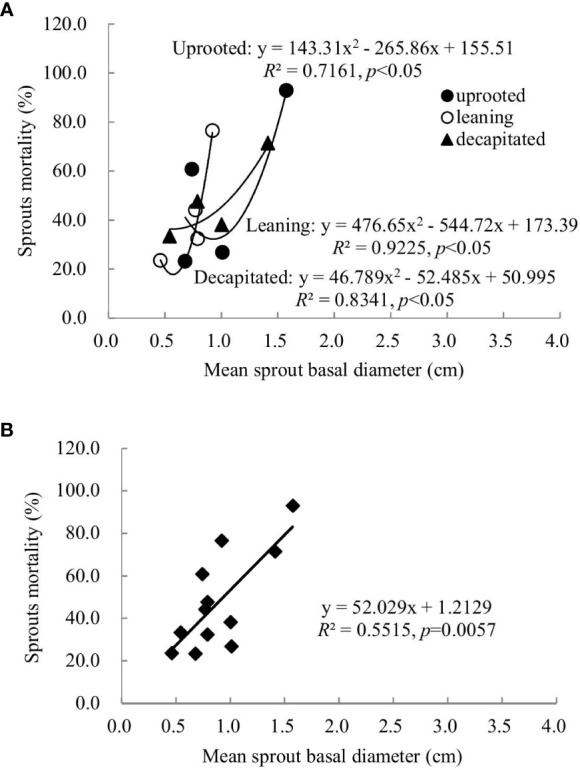
The relationship between the sprout basal diameters and the sprout mortality rates of sprouts germinated in 2009 over 5 years of recovery from damage. **(A)** Uprooted trees, leaning trees, and decapitated trees and **(B)** total individuals of all damaged trees were analyzed.

**Figure 7:**
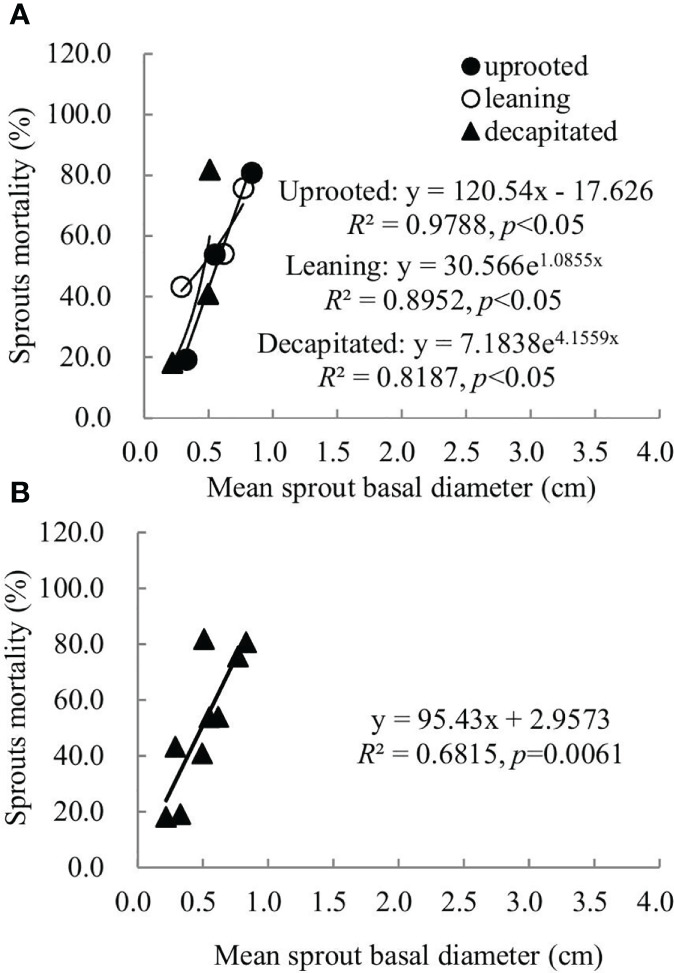
The relationship between the means of sprout basal diameters and the means of sprout mortality rates of the sprouts germinated in 2010 over 4 years of recovery from damage. **(A)** Uprooted trees, leaning trees, and decapitated trees and **(B)** total individuals of all damaged trees were analyzed.

Sprouts that boomed in the second and third years after the disaster (2009 and 2010) exhibited a linear positive correlation between the diameters and cumulative mortality rates during the recovery period (*R* ^2^ ranged from 0.5515 to 0.6815). In other words, the annual increase in the sprout diameters was positively correlated with the cumulative mortality rates of these sprouts (**Figures 6**, **7**). For the sprouts produced in 2009 on the uprooted, leaning, and decapitated trees, there were significant quadratic polynomial relationships between the sprout diameters and the cumulative mortality rates during the recovery period (**Figures 6A, B**, *p*< 0.05). Among them, the cumulative mortality rates of sprouts of the uprooted and leaning trees decreased at first and then increased along with the sprout diameters, while sprouts of decapitated trees only exhibited a positive correlation between cumulative mortality rates and sprout diameter (**Figure 6**). For the sprouts that boomed in 2010 on the uprooted trees, there was a significant linear positive correlation between the sprout diameters and their cumulative mortality rates (**Figure 7**, *R* ^2 = ^0.9788, *p*< 0.05). Significant power function relationships were found in the sprouts from leaning and decapitated trees (*p*< 0.05).

## 4 Discussion

### 4.1 Sprouts produced in the early years after disasters are of high energy reserve and survival rates

Many species produced more sprouts in the first growing season after cutting than those produced later (Imanishi et al., 2010; Zhu et al., 2012; Xue et al., 2013; Dinh et al., 2018). The number of living sprouts tends to decline in the following years (Rijks et al., 1998). In another 6-year study, sprouting rates in boreal places (Li et al., 2013) exhibited the same trend as our study. This indicates that subtropical trees may share the same recovery responses to damages. Sprouting number has been found to positively correlate to damage severity on trees (Wang et al., 2016). It is possible that enhanced shoot sprouting on trees after damage may help to maintain their “ecological niche” in forests (Clarke et al., 2013; Cao et al., 2020). Climate changes resulted in an increased frequency of extreme events. Studying the recovery of trees damaged in natural calamities would provide more reference for forest management. As studies on a subtropical area are rare, our study here could serve as a reference.

The mortality and growth of sprouts are dependent on tree conditions before being damaged. Tree sizes and energy reserves were two key factors, which mainly determine the initial sprouting capacity, sprout vigor, and mortality (Moreira et al., 2009). As sprouting is an energy-consuming process, it is reasonable that more sprouts boomed in the early years when the energy reserves are adequate and then decreased year by year along with energy consumption (Cao et al., 2020). Resprouting vigor decreased when reserves were depleted (Iwasa and Kubo, 1997; Zhu et al., 2012). Thus, resprouting mortality increased (Moreno and Oechel, 1991). Although these principles are clear, the long-term dynamics of sprout mortality has not been addressed in detail (Moreira et al., 2009). Especially, information on subtropical trees is largely unknown. Previous studies investigated the relationship between tree size and sprouting for a short period or compared the difference of sprouts on different tree species (Paciorek et al., 2000; Dinh et al., 2018). Our study recorded the mortality of *S. superba* for 6 years, which provided sound evidence that supported the theory above. Furthermore, *S. superba* is a subtropical evergreen plant. This study indicated that sprouting responses to disasters of subtropical trees are similar to the trees in temperate or boreal areas.

### 4.2 The dynamics of sprout mortality rates plays an important role in forest recovery from damage

The importance of resprouting for forest restoration has been observed for a long time. However, factors influencing sprout mortality are not well modeled. Paciorek et al. (2000) found that resprouting rate and mortality rate were varied between forest tree species. Ladwig et al. (2019) reported that creosote bush [*Larrea tridentata* (DC.) Coville] had a sprouting rate as high as 99% in the first year after an extremely cold winter. Accordingly, the recovery speed of creosote bush was quicker, and canopies had recovered up to 83% of the original canopy sizes before the extreme freezing winter (Ladwig et al., 2019). Sprouts produced in different years are of different survival rates. Our results on *S. superba* and also other trees reported in previous studies consistently showed that the early sprouts are of higher survival rates than the late ones (Dinh et al., 2018). Thus, management of resprouting in the early years is more important for forest recovery from damage. Sprouting positions of damaged trees also have a big influence on sprout mortality (Dinh et al., 2018). Previous studies reported that sprouts originating from stems had greater mortality than those growing from root crowns (Neke et al., 2006).

In our study, we found that survival rates were affected by sprout position and were also dependent on the types of damaged trees. For the decapitated trees, sprouts on higher positions exhibited a higher survival rate, while for uprooted or leaning trees, sprouts at the bases of trunks had a higher survival rate (**Figures 5** **–** **7**). In addition, we also found that some common positions among the three damaged types were also good for sprouting (**Figures 5** **–** **7**). These findings could guide practical applications in forest management in order to increase the resilience of these ecosystems and reduce the negative economic or ecological impacts of disturbances. In the future, we will try our best to keep the early branch sprouts of the decapitated trees, to promote their recovery and growth and form the canopy early.

To date, it is not clear which factors mostly influence the accumulative mortality rate of new sprouts during recovery from disturbances like ice storms or hurricanes. This study explored whether there was a correlation between the accumulative mortality rate of new sprouts and the average diameter of new sprouts that boomed in each year (**Figures 5** **–** **7**), providing a theoretical basis for the quantitative evaluation of the mortality rate of sprouts. In our research, whether there is a linear or non-linear correlation between the accumulative mortality rate and the average diameter is affected by different damage types. Large-scale follow-up of resprouting is difficult, which hinders broad-scale comparative studies (Jaureguiberry et al., 2020). Our study provides a simple and feasible method for the construction of a sprout regeneration model or understanding of the renewal dynamics of damaged trees under future climate change.

### 4.3 Practical management for the sprouts of damaged trees

Natural disturbances play a key role in ecosystem dynamics, which influence forest management (Seidl et al., 2011). Under climate change and disturbance conditions, studying current forests also provides an opportunity to check which forests have proved to be the most and the least important in terms of resisting disturbance and climate change (Achim et al., 2022). Consideration should be given to managing post-disturbance events to achieve potential key benefits of sustainable forest management, such as the establishment of regeneration (Achim et al., 2022). The mortality and regeneration of damaged trees should be assessed to identify the main factors affecting short- to medium-term post-disturbance recovery (Roula et al., 2020). This successional survey provided a recovery profile of the subtropical tree *S. superba*, which could be beneficial to predicting tree response to disturbance in the subtropical areas. In our study, information about the dynamic relationships and the linear or non-linear correlations among sprout mortality rates, sprout diameter, and damaged types could be made into a model that provides a useful and convenient decision-making tool for forest managers, such as the model made to optimize the salvage harvest of forest farms after a fire (Kwon et al., 2021). Different management measures according to the capacity of sprouts could be considered during the recovery process. In practice, basal sprouts of uprooted trees should be kept to improve the restoration process in the broadleaved damaged forest.

The increase in sprout mortality in the later years may be associated with self-thinning. Xue et al. (2013) found that self-thinning started after 2 years of cutting in Chinese cork oak (*Quercus variabilis* Bl.). Self-thinning in the sprouts of *S. superba* started in the third year after the ice storm. A higher mortality rate appeared in the later years (**Figures 2** **–** **4**). Therefore, sprout number should be adjusted in the first 3 years after an ice storm for the management of damaged *S. superba* forests. Self-thinning is a strategy to avoid energy wasting to enhance the growth of living sprouts (Xue et al., 2013; Keyser and Zarnoch, 2014; Montoro Girona et al., 2019; Montoro Girona et al., 2023b). Consistently, sprout thinning has been applied on trees after cutting or post-fire, which is considered sustainable management for forests (Montoro Girona et al., 2019; Roula et al., 2020). We also studied the position effects on sprout vigor. Practically, large sprouts on the top of decapitated trees should be kept, as they are of the highest vigor and more likely could grow to large branches. In future research about the impact of an ice storm or windthrow on regeneration, sprout growth should be investigated continuously to provide more theoretical support for the construction of a mechanism model for the regeneration process after disturbances like windthrow (Gardiner et al., 2008; Kwon et al., 2021).

## 5 Conclusions

In this study, we have recorded the sprout mortality of *S. superba* for 6 years after an ice storm. It was found that sprout mortality was increasing year by year. The sprout position on damaged trees also affected sprout mortality. A linear or non-linear correlation between the accumulative mortality rates and the sprout diameters was analyzed. The sprout survival rate of *S. superba* was found to be dependent on the types of damaged trees and sprout numbers in the first year after damage. These results provided a valuable reference for the management of subtropical forests, which could be used by forest managers and scientists. We point out that it is better to keep the sprouts that boomed in the early years and prune the late sprouts in order to promote forest recovery from damage. In the future, it is necessary to study the correlation between sprout survival rates and sizes of damaged trees. A long-term study would provide more information on both theoretical and practical values for forest management.

## Data availability statement

The original contributions presented in the study are included in the article/supplementary material. Further inquiries can be directed to the corresponding author.

## Author contributions

Conceptualization, YC; methodology, YC and BZ; software, YC; validation, YC; formal analysis, YC; investigation, YC, XW and BZ; resources, YC; data curation, YC; writing—original draft preparation, YC; writing—review and editing, YC; visualization, YC; supervision, YC; project administration, YC and BZ; funding acquisition, YC and BZ All authors have read and agreed to the published version of the manuscript.
